# Development and validation of a food and nutrition literacy questionnaire for Chinese school-age children

**DOI:** 10.1371/journal.pone.0244197

**Published:** 2021-01-06

**Authors:** Tan Liu, Xiao Su, Niuniu Li, Jing Sun, Guansheng Ma, Wenli Zhu

**Affiliations:** Department of Nutrition and Food Hygiene, Peking University School of Public Health, Beijing, China; Addis Ababa University, ETHIOPIA

## Abstract

**Background:**

This study aimed to develop and validate the Food and Nutrition Literacy Questionnaire for Chinese School-age Children (FNLQ-SC).

**Methods:**

A comprehensive literature review and qualitative study were initially performed to identify the dimensions and core components of food and nutrition literacy. A cross-sectional survey of 4359 school-age children was conducted, and junior middle school students were used to analyze the reliability and validity of the questionnaire (n = 2452). The reliability of the questionnaire was determined by internal consistency, the construct validity was assessed by exploratory factor analysis (EFA) and confirmatory factor analysis (CFA), and the content validity was assessed by the Pearson correlation coefficient.

**Results:**

From the literature review and qualitative methods, 19 core components of the FNLQ-SC were developed, including one dimension of food and nutrition knowledge and understanding, and four skill dimensions (ability of access, selection, preparing food and healthy eating). The overall FNLQ-SC questionnaire had acceptable internal consistency (Cronbach’s α = 0.698). The EFA of skill components extracted 5 factors that were included in the conceptual framework in a slightly different model, and the cumulative contribution of variance accounted for 50.60% of the overall variance. The CFA of skill components showed an acceptable fit in general and the root mean square error of approximation (RMSEA) was 0.070 (between 0.50 to 0.80). The communality was greater than 0.20 for all components. The Pearson correlation coefficients between each dimension and the overall questionnaire ranged from 0.370 to 0.877. The average FNLQ-SC score of all 4395 participants was 61.91 ± 9.22, and the score for the knowledge and understanding dimension was higher than that for the skill dimensions. Multiple linear regression analysis indicated that not only social demographic characteristics (being a girl, being an only child, living someplace other than at school, having an urban registered permanent residence status, being from an affluent family, and being cared for by parents/grandparents with a higher education level) but also the home food environment were predictors of food and nutrition literacy in school-age children (R^2^ = 0.226, *F* = 81.401, *P*<0.05).

**Conclusion:**

The Food and Nutrition Literacy Questionnaire (FNLQ-SC) developed here had good reliability, and it can potentially be a useful instrument for assessing food and nutrition literacy among Chinese school-age children.

## Introduction

The Global Burden of Disease Study 2017 indicated that dietary risks were responsible for 22% of all deaths and 15% of all disability adjusted of life years (DALYs) among adults globally, and these numbers were even higher in China (30.2% and 21.3%, respectively) [1]. In addition, the triple burden of malnutrition (undernutrition, micronutrient deficiencies and overweight) threatens the survival, growth and development of children and is driven by the poor dietary quality [2]. The Global School-Based Student Health Survey (GSHS) in 2019 showed roughly half of the countries reported that 10%-30% of 13–15 years old students did not eat any fruit at all, and a quarter reported that 10%-30% of students did not eat any vegetables at all; Nearly 70% of countries reported that at least half of their students eat fast food on a weekly basis; all countries found that one out of five students consumed carbonated soft drinks at least once a day [3].

Improving the dietary habits of children is a multifaceted societal task that demands an understanding of the social context and individual food-related skills and abilities [4]. At individual level, food and nutrition literacy, which is the capacity to obtain, process, and understand nutrition information and the skills needed to make appropriate nutrition decisions and maintain a healthy diet, has become an increasingly important concept [5, 6]. A systematic review showed that food literacy may play a role in shaping adolescent’s dietary intake, in addition, findings suggest that food skills and behaviors learned in adolescence are sustained later in life [7]. Improving children’s food and nutrition literacy has been a particular target of intervention studies and contemporary nutrition policies and action [8].

There are several instruments for measuring food and nutrition literacy, such as the Nutrition Literacy Assessment Instrument (NLAI) [9], the Nutrition Literacy Scale (NLS) [10], the Critical Nutrition Literacy Scale (CNL) [11], the Newest Vital Sign (NVS) [12], and the Food and Nutrition Literacy Questionnaire (FNLIT) [13]. Most of these instruments were developed for assessment in adults, except the FNLIT, which was created for use with Iranian school-age children. Considering the dietary culture gaps between different countries and the cognition differences between adults and children, the above instruments cannot be used for assessing Chinese children.

Overall, food choices and dietary quality in childhood can affect the lifelong risk of nutrition-related diseases [14, 15]. According to studies, adequate nutrition knowledge, optimal dietary behaviors, and the maintenance of a healthy weight are now recognized as key modifiable factors in health promotion and chronic disease prevention [16, 17]. Food and nutrition literacy level is one way to understand the reasons behind nutrition-related problems and behaviors among children and adolescents [7]. However, there are no food and nutrition literacy assessment instruments specifically developed and validated for Chinese school-age children.

In China the health literacy among residents has been monitored nationwide from 2008. While the health literacy assessment instrument can reliably identify individuals with health literacy skills, it is not specific to nutrition. This distinction is important because literacy is situation specific, and someone’s capacity may be perfectly adequate in one setting and marginal or inadequate in another. Functional literacy is situation specific, and the instruments for measuring Chinese health literacy are likely inadequate to measure food and nutrition literacy.

Our study aimed to develop and validate the Food and Nutrition Literacy Questionnaire for Chinese School-age Children (FNLQ-SC) to assess the food and nutrition capacity of children and provide targets for further nutrition education and intervention.

## Materials and methods

### Development of questionnaire

The development of the Food and Nutrition Literacy Questionnaire for Chinese School-age Children (FNLQ-SC) mainly comprised two stages:

#### Stage 1: The construction of food and nutrition literacy core components for school-age children

First, the conceptual framework and dimensions of food and nutrition literacy in school-age children were preliminarily constructed based on a literature review and expert interview, considering the cognitive level and dietary behavior problems of school-age children. A systematic retrieval of the literature was performed using the key words of “food literacy, nutrition literacy” from the earliest data to June 2018, in the English and Chinese databases of PubMed, Web of Science, ScienceDirect, CNKI and WanFang. And additional publications were identified by conducting a hand search of references in included publications. Then an expert panel meeting was convened in August 2018, to discuss face to face and develop a provisional framework of food and nutrition literacy of school-age children. The experts were qualified with adequate experience in nutrition, health education and primary education, who would participate in the following Delph consultation, in which their detailed information would be presented.

In this study, food and nutrition literacy is defined as a collection of interrelated knowledge, skills and behaviors required to plan, manage, select, prepare and eat foods to meet needs and determine food intake [18]. According to Nutbeam’s hierarchical model, food and nutrition literacy can be classified into three levels: functional, interactive and critical literacy [19]. Functional literacy is the ability to obtain, understand and use information on food and nutrition, including knowledge on various food and nutrition topics, and the practical skills needed to obtain, select, prepare and eat healthy foods. Interactive literacy is the ability to exchange, share, discuss information on food and nutrition with others and participate in shared actions [6]. Critical literacy is the ability to judge food and nutrition information critically, recognize the influence of nutrition and food decisions on the society, understand food as integrative part of a complex production and distribution process, and recognize the influence of different social conditions on food choice and dietary behavior [6, 13]. At the same time, we also referred to the evidence-based Chinese Dietary Guidelines (2016) as a behavior blueprint.

Second, a qualitative consensus study was conducted to determine the dimensions and core components of the FNLQ-SC. A two-stage electronically distributed Delphi consultation was held with 15 food and education experts, based on the following criteria: 1) experts with adequate experience in nutrition, health education and primary education; 2) representatives of Chinese nutrition society. The Delphi study was conducted from September 2018 to December 2018, which consisted of two rounds. In the first-round survey a Delphi questionnaire with the outline of a provisional food and nutrition literacy core components was mailed to each expert. Each member was asked to rate the appropriateness of each statement using a five-point Likert-type scale (1-unnecessary; 2-unimportant; 3-less important; 4-important; and 5-necessary). A consensus in this study was defined a priori when agreement (4–5) was provided by a minimum of 75% of the experts. A summary of the first-round survey was discussed by our research team and the FNL components was revised. Then we implemented a second-round survey using the same method as well as the revised components, until a compromise was reached.

#### Stage 2: Questionnaire development

Based on the conceptual framework identified at stage one, a pool of 51 questions was generated to measure the core components of food and nutrition literacy. The questions included 5-point Likert-type questions (“I am concerned about nutrition and health information: never, seldom, sometimes, usually, always.”), choice questions (“Which of the following snacks is healthier?”), and fill-in-the-blank questions (“Fill in your height and weight.”). Because the questionnaire was developed with the children’s real-life situation in mind, sometimes one question assessed more than one component of food and nutrition literacy. Therefore, the reliability and validity were analyzed based on the components, not the questions.

The appropriateness of the questionnaire was evaluated by food and nutrition experts in the study steering group, and the readability and difficulty of the questionnaire were evaluated and adjusted by two senior teachers in primary and junior middle schools. After redundant items were eliminated, the final questionnaire included 50 questions.

### Validation of questionnaire

#### Data collection

From Baoding district, Hebei Province of China, five intermediate level schools (three primary and two middle schools) were selected as investigation sites, according to regional socioeconomic and student’s population density.

The participant students were voluntarily recruited in June 2019. The inclusion criteria of participant students were as follows: being apparently healthy without severe acute or chronic physical and mental diseases, being able to fill out the self-administrated questionnaire. And the students who did not meet the inclusion criteria were excluded. Based on this, the investigators explained the investigation protocol to all 4520 students from grade 3 to 8 and their parents or guardians while parent-teacher conference. Finally, written informed consent was obtained from 4359 children and their guardians, the response rate was 96.4%. Even though there was not formula calculating, the sample size was sufficient to draw conclusion referring to literatures [20–25].

For all participants, food and nutrition literacy was assessed using the FNLQ-SC, and social demographic characteristics (age, sex, registered residence, family affluence status, caregivers and their education levels), home food environment, and school nutrition education were investigated by a self-reported questionnaire. Family affluence status was assessed using the adjusted Family Affluence Scale (FAS), which is a six-item scale that was used in a WHO collaborative cross-national study of the Health Behavior in School-aged Children (HBSC) [26]. Considering the Chinese family situation, three items were used to assess family affluence status in the study: “1) Does your family own a car, van or truck? 2) Do you have your own bedroom for yourself? 3) How many times did your family travel for a holiday/vacation last year?” The home food environment construct comprised the healthy food (fruit) accessibility at home, family food rules (Parents try to get me to eat more food), family eating behavior (eating out, limiting screen activity while mealtime), and discussion of nutrition information with families.

The study protocol was approved by the Peking University Institutional Review Board (Beijing, China, approval number RB00001052-17115), and conducted according to the Declaration of Helsinki and ethical guidelines. The privacy of participant students and the confidentiality of their personal information would be protected.

#### Reliability tests

Several psychometric properties of the FNLQ-SC were assessed. To reduce the effects of cognition difference, only the junior middle school student samples were used to analyze the reliability and validity of the questionnaire (n = 2452).

The internal consistency reliability was measured by calculating the Cronbach’s alpha coefficient of the overall questionnaire as well as of each dimension and each component. For the overall questionnaire, a coefficient greater than 0.7 indicated acceptable reliability [20–22].

The floor and ceiling effects were assessed by the proportion of respondents who had the lowest or highest score [23].

#### Validity tests

To assess the construct validity of the scale, exploratory factor analysis (EFA) was used to explore whether the statements in the questionnaire reflected the conceptual framework of the FNLQ-SC. Considering that the knowledge and skill dimensions were based on different logical frameworks and there was only one dimension in cognitive domain, we analyzed only the components of the skill domain by EFA. The Kaiser-Meyer-Olkin (KMO) measure (>0.7) was used to determine the sampling adequacy. Bartlett’s test of sphericity (*P*<0.05) and total variance explained were used for the evaluation of factor analysis. Oblique rotation and principal axis factoring (PAF) extraction were used to explore the existing factorial pattern. The number of factors was determined through eigenvalues above 1, the percent of explained variance by each factor, scree plot and interpretability criteria. Confirmatory factor analysis (CFA) was also conducted for the skill domain, and the root mean square error of approximation (RMSEA) being equal or smaller to 0.08 was considered an acceptable fit (≤0.05 as a good fit). Two incremental fit indexes were selected, the goodness of fit index (GFI) and adjusted goodness of fit index (AGFI), which values at or above 0.90 were considered a good fit.

The content validity was assessed by the Pearson correlation coefficients between the components, dimensions and the overall questionnaire. The coefficient more than 0.6 suggested the components and dimension had good discrimination and correlation with overall index.

#### Statistical analysis

Internal consistency and other parametric tests were computed by using SPSS 25.0. The significance level was set at *P*<0.05.

The reliability and validity were analyzed on the basis of components, not the questions, because some questions assessed more than one components.

The questionnaire consisted of 50 questions, and each question was scored 2 points. The students of grades 7–8 were assessed using the full FNLQ-SC and therefore had a score of 100, while the students of grades 5–6 skipped one question and had a full score of 98, and the students of graders 3–4 skipped 4 questions and had a full score of 92. The final score was converted into a centesimal measure for comparison.

The dimensions of knowledge and understanding, access to and planning for food, selecting food, preparing food and eating included 15, 5, 5, 10, 15 questions respectively. In order to compare among groups the final score was converted into a centesimal measure for each dimension.

## Results

### Core components of food and nutrition literacy in school-age children

A total of 25 core components of food and nutrition literacy in school-age children were originally proposed. An electronically distributed two-round Delphi consultation was conducted. The response rates of both rounds were 100%, and the average authority degree of the experts was 0.8767. The mean of the total scores (4.60) for all components in the second round was better than that in the first round (4.38), and the total coordination coefficient was 0.22 in the first round and 0.18 in the second round (both *P* < 0.001). Finally, 19 core components of the FNLQ-SC were determined, including one dimension of food and nutrition knowledge and understanding, and four skill dimensions (ability of access, selection, preparing food and healthy eating), as well as three levels of functional, interactive and critical literacy, as shown in Table 1.

**Table 1:** The core components of food and nutrition literacy in school-age children.

Domain	Dimension	Component	The 2^nd^ round consultation	
			Average	coordination coefficient
			score	
Knowledge and understanding	Knowledge and understanding of food and nutrition	1. Understanding that an individual is responsible for his or her own health and lifestyle.^a^	4.67	0.10
		2. Knowing about food and nutrition information sources and services.^a^	4.20	0.24
		3. Understanding the food system from production to access to waste.	4.32	0.18
		4. Knowing about food groups and their compositions.^a^	4.43	0.23
		5. Understanding a variety of dietary cultures.^a^	3.91	0.20
Skill	Access to and planning for food	6. Learning to grow food in the garden and process homegrown food.^a^	4.60	0.18
		7. Planning the quantity of food to prepare to reduce food waste.^a^	4.17	0.13
	Selecting food	8. Being able to judge the quality of food.^a^	4.39	0.24
		9. Being able to read and understand food nutrition labels.^a^	4.60	0.16
		10. Being able to critically judge advertisements, promotions, marketing and other information presented to consumers.^c^	4.20	0.24
		11. Talking to families and friends about food and nutrition, saying “no”, and being able to modify their intake.^b^	4.40	0.21
	Preparing food	12. Being familiar with kitchen equipment and being able to help parents prepare and cook foods.^a^	3.99	0.19
		13. Being able to apply basic principles of food safety, like keeping one’s hands clean.^a^	4.38	0.23
	Eating	14. Being able to estimate food portion size.^a^	4.43	0.16
		15. Healthy and balanced diet, including vegetables and fruits, dairy and legume products, whole grains and fewer oils, salts and sugars.^a^	4.62	0.14
		16. No picky eating.^a^	4.60	0.11
		17. Eating snacks healthily.^a^	4.27	0.19
		18. Measuring and evaluating weight regularly, maintaining a healthy weight by regulate energy balance between dietary intake and physical activity expenditure. ^a^	4.64	0.14
		19. Abiding by table manners, and chewing food thoroughly. ^a^	4.02	0.23

^a^ Functional literacy.

^b^ Interactive literacy.

^c^ Critical literacy.

### Demographic characteristics of participants

A total of 4359 students in grades 3–8 participated in the study, including 2195 boys (50.36%) and 2105 girls (48.29%). Among those, the junior middle school student samples (n = 2452) were used to analyze the reliability and validity of the questionnaire, and the total samples (n = 4359) were used for the final study. The sociodemographic characteristics of the two study samples are shown in Table 2.

**Table 2:** Demographic characteristics of participants, n (%).

Characteristics	Total (N = 4359)	Reliability and validity study (N = 2452)
**Sex**		
Male	2195 (50.36)	1213 (49.47)
Female	2105 (48.29)	1216 (49.59)
**Age (years)**		
7~9	237 (5.44)	15 (0.61)
10~12	1511 (34.66)	70 (2.85)
13~15	2492 (57.17)	2270 (92.58)
16~17	61 (1.40)	61 (2.49)
**Grade**		
3~4	853 (19.57)	—
5~6	1054 (24.18)	—
7~8	2452 (56.25)	2452 (100.00)
**Only child**		
Yes	1003 (23.01)	520 (21.21)
No	3320 (76.16)	1919 (78.26)
**Living at school**		
Yes	1134 (26.02)	1086 (44.29)
No	3194 (73.27)	1354 (55.22)
**Registered residence**		
Urban	1643 (37.69)	878 (35.81)
Rural	2616 (60.01)	1520 (61.99)
Family affluence status*		
Poor (≤2)	572 (13.12)	334 (13.62)
Medium (3–5)	2183 (50.08)	1306 (53.26)
Affluent (6–7)	1533 (35.17)	788 (32.14)
**Principal caregiver**		
Parents	3430 (78.69)	1948 (79.45)
Grandparents	722 (16.56)	392 (15.99)
Other*	129 (2.96)	65 (2.65)
**Caregiver’s educational level**		
Primary school or below	514 (11.79)	220 (8.97)
Junior high school	1733 (39.76)	1203 (49.06)
Senior high school or equivalent	890 (20.42)	451 (18.39)
Junior college	340 (7.80)	137 (5.59)
Bachelor’s degree or higher	286 (6.56)	126 (5.14)
**School nutrition education**		
Yes	2534 (58.13)	1296 (52.85)
No	1761 (40.40)	1132 (46.17)

Note: The sum of percentages did not add up to 100.00% because of the default value.

* Other caregivers include siblings, babysitters and all other caregivers, except the parents and grandparents of children.

### Reliability

The overall FNLQ-SC questionnaire had acceptable internal consistency (Cronbach’s α = 0.698). The Cronbach’s α coefficients for the five dimensions (knowledge and understanding, access to and planning for food, selecting food, preparing food, eating), were 0.452, 0.300, 0.244, 0.148, and 0.436, respectively.

An additional alpha test that deleted components one at a time showed that removing components did not result in an increase in the Cronbach’s alpha, showing that each component had acceptable internal consistency with the overall questionnaire, as seen in Table 3.

**Table 3:** Factor analysis results and component analysis of the FNLQ-SC.

Component	EFA factor loading					Communality	α if component deleted	Pearson correlation coefficient
	Factor 1	Factor 2	Factor 3	Factor 4	Factor 5			
1						0.450	0.631	0.418
2						0.469	0.632	0.372
3						0.337	0.635	0.283
4						0.382	0.626	0.453
5						0.349	0.631	0.376
6	-0.037	0.709*	0.111	0.253	-0.014	0.503	0.633	0.320
7	0.450	0.461	0.092	-0.159	-0.137	0.427	0.633	0.371
8	0.629*	-0.141	0.016	-0.030	0.041	0.392	0.636	0.289
9	0.109	0.036	0.690*	-0.043	0.113	0.437	0.616	0.504
10	0.278	-0.260	0.032	0.434	-0.304	0.491	0.645	0.114
11	0.144	0.311	0.420	-0.140	0.285	0.335	0.620	0.456
12	0.113	0.752*	-0.019	-0.089	0.000	0.374	0.637	0.266
13	0.395	0.045	0.202	-0.375	-0.417	0.416	0.637	0.299
14	-0.060	-0.048	0.665*	0.024	-0.124	0.416	0.636	0.294
15	0.634*	0.176	0.180	0.291	0.142	0.574	0.607	0.720
16	0.085	0.101	0.089	0.743*	-0.006	0.586	0.636	0.292
17	0.168	-0.069	0.076	-0.064	0.799*	0.474	0.641	0.239
18	0.114	0.076	0.539*	0.165	0.003	0.303	0.624	0.496
19	0.678*	0.270	0.052	0.153	0.062	0.545	0.610	0.535

Note: Only skill components were analyzed by EFA.

*Factor loading > 0.5.

CFA indicators of the skill domain showed an acceptable fit in general. The RMSEA was 0.070 (between 0.50 to 0.80), and GFI and AGFI were close to 0.90, being 0.838 and 0.813 respectively.

### Construct validity

Only 14 components of the skill dimensions were analyzed by EFA. The KMO test showed sampling adequacy (KMO = 0.738), and Bartlett’s test confirmed that factor analysis was appropriate (*P*<0.001). Finally, EFA extracted 5 factors with eigenvalues greater than 1, and the cumulative contribution of variance accounted for 50.60% of the overall variance. The model identified five factors as “Factor 1, selecting and eating”, “Factor 2, access and preparation”, “Factor 3, food labels and measurements”, “Factor 4, picky eating”, and “Factor 5, eating snacks”. The communality was more than 0.20 for all components, as shown in Table 3.

### Content validity

The Pearson correlation coefficients between different dimensions ranged from 0.152~0.400. The correlation coefficients between each dimension and the overall questionnaire ranged from 0.370 to 0.877, especially the coefficients of dimensions of knowledge and understanding, selecting food, and eating, were more than 0.6, which showed a strong correlation with the overall questionnaire (Table 4).

**Table 4:** Pearson correlation coefficient among dimensions of FNLQ-SC.

Dimensions	Knowledge and understanding	Access to and planning for food	Selecting food	Preparing food	Eating	Total
Knowledge and understanding	—	0.241	0.326	0.167	0.400	0.658
Access to and planning for food	0.241	—	0.214	0.257	0.303	0.441
Selecting food	0.326	0.214	—	0.152	0.369	0.657
Preparing food	0.167	0.257	0.152	—	0.188	0.370
Eating	0.400	0.303	0.369	0.188	—	0.877

The Pearson correlation coefficients between each component and the overall questionnaire ranged from 0.114 to 0.504, and the coefficients of eight components were less than 0.3, as shown in the last column of Table 3.

### Assessing food and nutrition literacy and its related factors in school-age children

According to the centesimal score, the average FNLQ-SC score of all participants was 61.91 ± 9.22, and participants’ scores ranged from 22.83 to 92.86. None of the respondents scored the maximum of 100 or the minimum of 0; therefore, neither floor nor ceiling effects likely occurred. Among the dimensions, the score for knowledge and understanding (64.78±15.15) was higher than the score for skill dimensions, and the score for eating was the lowest (60.45±11.00).

As shown in Table 5, the children who were girls, were only children, did not live at school, had an urban registered permanent residence, belonged to an affluent family, were cared for by their parents/grandparents with higher education levels, and had nutrition education experience in school had significantly higher food and nutrition literacy (*P*<0.05).

**Table 5:** Distribution of food and nutrition literacy in school-age children (n = 4359, mean ± SD).

Variables	Total		Knowledge and understanding	Access to and planning for food	Selecting food	Preparing food	Eating
**Total**	61.91±9.22		64.78±15.15	61.42±21.28	63.43±14.22	62.03±13.05	60.45±11.00
Sex							
Male	61.28±9.48^a^		64.81±15.37	59.72±21.65^a^	62.40±14.60^a^	61.51±13.55^a^	59.84±11.35^a^
Female	62.72±8.80^b^		64.99±14.74	63.20±20.74^b^	64.66±13.65^b^	62.56±12.41^b^	61.25±10.50^b^
Age (years)							
7~9	61.55±9.65^a^		62.28±16.59^a^	61.13±20.54^ab^	62.03±15.93^a^	60.38±14.48^a^	61.44±11.13^a^
10~12	63.39±9.63^b^		65.65±14.94^b^	63.79±22.54^a^	64.25±14.87^b^	61.70±13.71^ab^	62.71±11.28^a^
13~15	61.30±8.77^ac^		64.86±14.89^b^	60.14±20.45^b^	63.27±13.59^ac^	62.45±12.40^b^	59.25±10.55^b^
16~17	56.58±7.29^d^		58.71±16.77^a^	60.25±21.95^ab^	58.24±11.89^ad^	60.82±13.91^ab^	54.10± 8.86^c^
Grade							
3~4	61.67±9.22^a^		62.06±15.61^a^	60.76±21.65^a^	63.13±16.57	60.84±14.56^a^	61.24±10.39^a^
5~6	63.94±9.80^b^		67.20±14.61^b^	66.02±22.53^b^	64.07±13.76	62.05±13.07^b^	63.19±11.77^b^
7~8	61.13±8.83^a^		64.69±15.06^c^	59.67±20.29^a^	63.25±13.50	62.43±12.45^b^	58.99±10.61^c^
Only child							
Yes	63.66±9.85^a^		66.58±15.44^a^	62.31±21.39	63.95±14.66	61.66±13.59	63.17±11.71^a^
No	61.46±8.91^b^		64.37±14.93^b^	61.17±21.23	63.35±14.03	62.16±12.82	59.70±10.62^b^
Living at school							
Yes	60.08±8.11^a^		63.66±14.83^a^	58.74±20.01^a^	64.35±13.13^a^	61.81±11.77	56.99±9.45^a^
No	62.62±9.47^b^		65.27±15.16^b^	62.36±21.66^b^	63.15±14.55^b^	62.09±13.45	61.73±11.22^b^
Registered residence							
Urban	64.15±9.48^a^		66.88±15.40^a^	63.51±21.72^a^	63.74±14.67	62.71±13.65^a^	63.82±11.15^a^
Rural	60.64±8.74^b^		63.70±14.69^b^	60.09±20.88^b^	63.36±13.86	61.62±12.62^b^	58.47±10.35^b^
Family affluence status							
Poor (≤2)	59.19±8.67^a^		61.16±14.99^a^	55.73±21.52^a^	62.70±14.26^a^	60.54±13.34^a^	57.19± 9.96^a^
Medium (3–5)	61.41±8.81^b^		64.79±14.81^b^	60.71±20.93^b^	63.16±14.14^a^	61.62±12.77^a^	59.68±10.60^a^
Affluent (6–7)	63.78±9.57^c^		66.37±15.25^c^	64.47±21.24^c^	64.30±14.20^b^	63.17±13.20^b^	62.87±11.39^b^
Principal caregiver							
Parents	62.14±9.11^a^		64.98±15.00^a^	61.65±21.01^a^	63.50±14.13^a^	62.30±12.85^a^	60.74±10.92^a^
Grandparents	61.77±9.14^a^		65.22±14.71^a^	60.79±21.75^a^	64.08±14.16^a^	61.10±13.16^b^	60.02±10.91^a^
Other*	57.68±10.66^b^		59.00±18.09^b^	57.07±25.62^b^	59.43±15.27^b^	59.92±16.04^c^	56.17±12.06^c^
Caregiver’s educational level							
≤Primary school	59.60±9.84^a^		62.79±15.16^a^	60.00±22.57^a^	61.05±15.61^a^	61.59±13.74^ab^	57.59±11.50^a^
Junior high school	61.13±8.40^b^		64.10±14.62^a^	60.04±20.00^a^	63.41±13.45^b^	61.40±12.60^a^	59.33±10.03^b^
Senior high school	63.80±9.11^c^		67.20±14.86^b^	62.87±22.81^b^	64.96±14.11^c^	61.71±13.43^ab^	62.80±10.82^c^
Junior college	64.85±9.75^c^		66.84±16.13^b^	64.67±20.80^b^	64.81±14.26^bc^	63.34±13.58^bc^	64.60±11.68^d^
≥Bachelor’s degree	66.93±9.51^d^		69.25±15.07^c^	65.12±21.48^b^	67.58±14.58^d^	64.76±12.17^c^	66.55±11.24^e^
School nutrition education							
Yes	63.54±8.85^a^		66.82±14.58^a^	63.78±20.85^a^	65.04±13.72^a^	62.63±12.93^a^	62.12±10.74^a^
No	59.71±9.16^b^		62.06±15.32^b^	58.14±21.47^b^	61.32±14.48^b^	61.21±13.03^b^	58.15±10.87^b^

Note: Different superscript characters (a, b, c, d, e) indicate significant differences among groups (*P<*0.05).

*Other caregivers include siblings, babysitters and all other caregivers except the parents and grandparents of the children.

Multiple linear regression analysis indicated that not only individual and family demographic characteristics but also the home food environment were predictors of food and nutrition literacy in school-age children (R^2^ = 0.226, *F* = 81.401, *P*<0.05), as shown in Table 6.

**Table 6:** Multiple linear regression analysis of food and nutrition literacy-related factors among school-age children.

Variables*	β	SE	B	*T*	*P*
Constant	38.304	1.494	—	25.632	<0.001
Sex	-1.215	0.275	-0.066	-4.415	<0.001
Age	-0.207	0.079	-0.040	-2.616	0.009
Only child	1.177	0.348	0.054	3.380	0.001
Registered residence	0.991	0.326	0.053	3.039	0.002
Family affluence status	0.357	0.086	0.067	4.174	<0.001
Principal caregiver					
Parents	—				
Grandparents	-0.254	0.370	-0.010	-0.687	0.492
Others	-3.020	0.825	-0.054	-3.659	<0.001
Caregiver’s educational level	0.961	0.147	0.113	6.525	<0.001
Accessibility of fruit at home	2.229	0.193	0.181	11.575	<0.001
Watching videos while eating at home	1.239	0.144	0.129	8.634	<0.001
Discussion nutrition information with families	1.509	0.144	0.159	10.493	<0.001
Family eating out	1.829	0.181	0.154	10.132	<0.001
School nutrition education	2.432	0.285	0.130	8.536	<0.001

Variable values: Sex (Male = 1, Female = 0); Only child (Yes = 1, No = 0); Registered residence (Urban = 1, Rural = 0); Caregiver’s educational level (Primary school or below = 1, Junior high school = 2, Senior high school or equivalence = 3, Junior college = 4, Bachelor’s degree or higher = 5); Home food environment variables such as “accessibility of fruit at home”, “Watching videos while eating at home”, “Discussion nutrition information with families”, and “Family eating out” were valued with the same options (Rarely = 1, Sometimes = 2, Often = 3, Always = 4); School nutrition education (Yes = 1, No/forgotten = 0).

## Discussion

Our study developed a questionnaire to assess food and nutrition literacy in Chinese school-age children. The questionnaire included five dimensions of knowledge and skill and 19 core components. The overall questionnaire had acceptable internal consistency (Cronbach’s α = 0.698). For the skill domain, the exploratory factor analysis (EFA) extracted 5 factors that were included in the conceptual framework but in a slightly different model, and the confirmatory factor analysis (CFA) showed an acceptable fit in general. The communality was more than 0.20 for all components. The Pearson correlation coefficients between the dimensions (knowledge and understanding, selecting food, eating) and the overall questionnaire were more than 0.6, which indicated a strong correlation. Using the questionnaire to assess food and nutrition literacy and its related factors in school-age children, the results showed that literacy was low; both social demographic characteristics and the home food environment were predictors of food and nutrition literacy in school-age children.

Improving dietary habits demands that individuals have food-related skills and abilities and requires an understanding of the social context. In this regard, nutritional science and education researchers are currently discussing the concepts of nutrition literacy and food literacy. A systematic review (2018) [6] revealed that nutrition literacy and food literacy are seen as specific forms of health literacy and represent distinct but complementary concepts. Definitions of nutrition literacy mainly describe the skills necessary to obtain and understand nutrition information, while food literacy incorporates a broader spectrum of theoretical and practical knowledge and skills to apply information on food choices, and critically reflect on the effect that food choice has on both personal health and society. Since food literacy is based on a more comprehensive understanding of health behaviors, the term is more appropriate to use in health promotion interventions. In this study, the term “food and nutrition literacy”, defined as a collection of interrelated knowledge, skills and behaviors required to plan, manage, select, prepare and eat foods to meet requirements and determine food intake, was used. We focused on not only the ability to access and understand nutrition information but also the ability to judge and apply nutrition information and the ability to communicate and act upon this information in the broader social environment to address nutritional barriers from personal, social, and global perspectives [11]. We developed the Food and Nutrition Literacy Questionnaire for Chinese School-age Children (FNLQ-SC) based on the conceptual framework using a literature review, expert interview and qualitative consensus study, which included five dimensions of food and nutrition knowledge; access, selection, preparation of food and healthy eating; and three levels of functional, interactive and critical literacy. The literature showed that all definitions of food and nutrition literacy contain elements of functional literacy, but only a few definitions describe interactive and critical literacy skills, this definition of which is based on Nutbeam’s model of health literacy [6, 11]. The core elements of all conceptual frameworks include practical knowledge and skills to regulate food intake, such as skills for planning meals and selecting and preparing food. The NLAI for American adults includes the following domains: appreciation of relationships between nutrition and health, knowledge of macronutrients, food measurement skill, numeracy and label reading, and skill in grouping like foods [9, 24]. Australian experts identified eleven components of food literacy grouped into four domains: planning and management, selection, preparation, and eating [18]. The Food and Nutrition Literacy (FNLIT) questionnaire [13] for elementary school children in Iran measures two domains with 6 subscales, including the cognitive domain (understanding and knowledge) and skill domain (functional, food choice, interactive, and critical skills). Overall, although the domain, dimensions and components vary with different food and nutrition definitions, the conceptual framework is similar, and future research should focus on multidimensional tools, including interactive and critical literacy, and the access, selection and preparation of food in addition to healthy eating.

We used the Cronbach’s α coefficient to analyze the internal consistency. The total Cronbach’s α was 0.698, which indicated that the overall questionnaire had acceptable internal consistency. However, the Cronbach’s α coefficient of various dimensions was low, ranging from 0.148 to 0.452. One possible explanation for the low internal consistency values of the dimensions is that internal consistency reliability values depend on the number of items in the scale [13]. Since the “planning”, “selection”, and “preparation of food” dimensions consisted of two, four and two components respectively, this may have caused the lower internal consistency values. Additionally, there are many other possible reasons for a low alpha value, such as poor interrelation between components and heterogeneous constructs [22], the sample size, and content overlap in different dimensions. However, the lower reliability estimates do not necessarily negate the value of the dimensions since the expert panel rated the components as relevant. Without evidence of acceptable internal consistency, we recommend that the total score be used instead of the subscale (dimension) scores.

Considering that the knowledge and skill dimensions are based on different logical frameworks, some studies have independently analyzed the variables of cognitive and skills domains by exploratory factor analysis (EFA) [13]. On the other hand, there was only one dimension in cognitive domain in this study, so only skill domain were analyzed, and 5 factors with eigenvalues greater than 1 were extracted, being “selecting and eating”, “access and preparation”, “food labels and measurements”, “picky eating”, and “eating snacks”. Compared with the conceptual framework of the study, factor of “access and preparation” equaled to the dimensions of “access to and planning for food” and “preparing food”, factor of “selecting and eating” equaled to the dimensions of “selecting food” and “eating”. Differently, factors of “picky eating” and “eating snacks” were combined in the “eating” dimension, and factor of “food labels and measurements” was included in the “selecting food” dimension. Overall, the EFA model was logically similar to the conceptual framework of the study, both according the food supply chain, despite a slight difference. And the CFA showed an acceptable but not good fit in general. Later we would modify or remove some questions to adjust the framework to fit well. Component analysis showed that the factor loading of some components was lower than 0.40, but the communality was more than 0.20 for all components. Removing any components did not result in an increase in the Cronbach’s alpha coefficient, which indicated that each component had acceptable internal consistency with the overall questionnaire. Additionally, in our study only Pearson correlations were analyzed statistically to measure the relations of each component with overall questionnaire, and between different dimensions, furtherly we will undertake logical validity by experts evaluation to calculate the content validity ratio (CVR) and content validity index (CVI). Besides, CFA would be used to evaluate the construct validity in another sample furtherly.

Using the FNLQ-SC, we assessed the food and nutrition literacy level of 4359 school-age children in grades 3–8. The results were similar to those of other studies. The FNLIT assessment of 803 students aged 10–12 years from elementary schools in Tehran, Iran, showed that more than half of the children (69%) had high levels in the cognitive domain of the FNLIT, but in the skills domain, very few (3%) scored highly [25]. Always the overall dietary quality index was used to determine the optimal FNL cut-off score [25], unfortunately the dietary intake was not investigated in our study, so the cut points of FNL could not been identified based on the subjects. Our study showed that the score of the knowledge dimension in school-age children was greater than that of skill dimensions. The FNLIT study identified some associations between the total FNLIT and its subscales and sociodemographic variables, including sex, parent’s education and age, and birth order. Their results indicated that girls felt more able than boys to exert choice and controlled over food and nutrition decisions but might be less able to do so in practice. Our results also showed that the total literacy in girls was higher than that of boys, but their critical literacy was lower than that of boys (*P*>0.05). Additionally, our results showed that the home food environment was significantly correlated with children's food and nutrition literacy. The total score of food and nutrition literacy was higher for the children who often had fruit at home, rarely ate out, did not eat in front of a screen, and frequently communicated about food and nutrition information with their families (*P*<0.05). Overall, these results are a general reminder to schools of the different learning needs of children from different family backgrounds: children in rural areas, younger children, children from large families, and children from families with a poor economic status and food environment should be the main target of nutrition education and nutrition improvement. The study highlights the need for continuous improvement in the nutrition education curriculum of schools in China, particularly highlighting the need for placing greater attention on the development of practical food and nutrition skills alongside more traditional food and nutrition knowledge. Additional studies are needed to more fully assess and understand the prediction capability of the FNLQ-SC.

## Conclusion

Overall, the FNLQ-SC has good reliability to some extent, and it could potentially be a useful instrument for assessing food and nutrition literacy in Chinese school-age children, despite the choice of convenience sampling and the risk of self-reported bias. It should be revised furtherly. The development and validation of an appropriate instrument is an essential step for food and nutrition literacy research in children. To our knowledge, this is the first reported food and nutrition literacy questionnaire for school-age children in China. The questionnaire can potentially be used with other Chinese populations. Of course, because the investigation sites and sample couldn’t represent Chinese school-age children, a nationwide survey of FNL was necessary to identify the target population for further nutrition education, to develop targeted interventions to improve the food and nutrition literacy and dietary quality of school-age children, thus further improving their health.

## Supporting information

S1 Table Corresponding of core components and FNLQ-SC questions. (DOCX)

S1 Dataset Minimal data set. (XLSX)

S1 File FNLQ-SC-Chinese. (PDF)

S2 File FNLQ-SC-English. (PDF)
