# Supplementary material for: Development and validation of a food and nutrition literacy questionnaire for Chinese school-age children
Source: PLoS One. 2021 Jan 6;16(1):e0244197. doi: 10.1371/journal.pone.0244197 (PMC7787443; doi:10.1371/journal.pone.0244197)
Supplement: S1 Table — (DOCX) [file pone.0244197.s001.docx]

**S1 Table. Corresponding of core components and FNLQ-SC questions**

| **Domain** | **Dimension** | **Component** | **Question number**  **in FNLQ-SC** |
| --- | --- | --- | --- |
| Knowledge and understanding | Knowledge and understanding of food and nutrition | 1. Understanding that an individual is responsible for his or her own health and lifestyle. | 1.(1) |
|  |  | 1. Knowing about food and nutrition information sources and services. | 1.(11) |
|  |  | 1. Understanding the food system from production to access to waste. | 4. |
|  |  | 1. Knowing about food groups and their compositions. | 5. 6. 14. |
|  |  | 1. Understanding a variety of dietary cultures. | 1.(3) |
| Skill | Access to and planning for food | 1. Learning to grow food in the garden and process homegrown food. | 1.(4) |
|  |  | 1. Planning the quantity of food to prepare to reduce food waste. | 1.(6) |
|  | Selecting food | 1. Being able to judge the quality of food. | 2.(7) |
|  |  | 1. Being able to read and understand food labels. | 1.(13) 13.(1)-(3) |
|  |  | 1. Being able to critically judge advertisements, promotions, marketing and other information presented to consumers. | 1.(10) |
|  |  | 1. Talking to families and friends about food and nutrition, saying “no”, and being able to moderate their intake. | 1.(2) 1.(12)  16. 17. |
|  | Preparing food | 1. Being familiar with kitchen equipment and being able to help parents prepare and cook foods. | 1.(7) |
|  |  | 1. Being able to apply basic principles of food safety, like keeping one’s hands clean. | 1.(8)  2.(8)-(10) |
|  | Eating | 1. Being able to estimate food portion size. | 15. |
|  |  | 1. Healthy and balanced diet, including vegetables and fruits, dairy and legume products, whole grains and fewer oils, salts and sugars. | 2.(2)(3)(5)(6)  3.(1)-(9) |
|  |  | 1. No picky eating. | 1.(9) 7. |
|  |  | 1. Eating snacks healthily. | 8. |
|  |  | 1. Measuring and evaluating weight regularly, maintaining a healthy weight by regulate energy balance between dietary intake and physical activity expenditure. | 9. 10. 11. 12. |
|  |  | 1. Abiding by table manners, and chewing food thoroughly. | 1.(5) 2.(1) 2.(4) |
