# Supplementary material for: Development and validation of a food and nutrition literacy questionnaire for Chinese school-age children
Source: PLoS One. 2021 Jan 6;16(1):e0244197. doi: 10.1371/journal.pone.0244197 (PMC7787443; doi:10.1371/journal.pone.0244197)
Supplement: S1 File — (PDF) [file pone.0244197.s003.pdf]

中国学龄儿童食物营养素养水平评价问卷(FNLQ-SC)

1. 根据自己的实际情况，在对应的序号处打√。

题目	非常不符合	不符合	一般	符合	非常符合
（1）我在选择食物时总会考虑是否营养。	①	②	③	④	⑤
（2）我通常自己决定吃什么食物。	①	②	③	④	⑤
（3）我对中国饮食文化很感兴趣。	①	②	③	④	⑤
（4）我会做一些中国传统美食（饺子、汤圆、粽子、春卷等）。	①	②	③	④	⑤
（5）我遵守餐桌礼仪（长者先、不喧闹、不乱翻饭菜、帮助收拾餐具等）。	①	②	③	④	⑤
（6）我吃饭的时候能吃多少就盛/买多少，很少浪费食物。	①	②	③	④	⑤
（7）我会帮助家长做饭（洗菜、摘菜、烹饪等）。	①	②	③	④	⑤
（8）我吃东西前洗手。	①	②	③	④	⑤
（9）我有两种及以上不爱吃且不吃的食物。	①	②	③	④	⑤
（10）我选择食物时会受电视、网络上食品广告的影响。	①	②	③	④	⑤
（11）我关注营养健康相关的信息。	①	②	③	④	⑤
（12）我会主动和别人讨论营养健康相关的信息。	①	②	③	④	⑤
（13）我购买食品时关注包装上的食品标签。	①	②	③	④	⑤

2. 你是否赞同以下行为？

情境	非常不赞同	不赞同	一般	赞同	非常赞同
（1）吃饭快慢对健康影响不大。	①	②	③	④	⑤
（2）早饭时间，还不饿就不吃了。	①	②	③	④	⑤
（3）不渴的时候不用喝水。	①	②	③	④	⑤
（4）一边吃饭一边看视频可以一举两得。	①	②	③	④	⑤
（5）果酒不算酒，可以喝。	①	②	③	④	⑤
（6）我正在长身体，要多多吃肉。	①	②	③	④	⑤
（7）仔细检查保质期过一天的面包，无异常就可以吃。	①	②	③	④	⑤
（8）剩饭要彻底加热后才能吃。	①	②	③	④	⑤
（9）没吃完的菜应及时放冰箱，不应等放凉。	①	②	③	④	⑤
（10）厨房切生肉的案板洗净后就能切熟肉。	①	②	③	④	⑤

3. 请根据你过去一周的实际情况，在下表对应的序号处打勾。

你过去一周内有几天吃了以下食物？	没有吃	1~2 天	3~4 天	5~6 天	每天都吃
（1）**粗粮**（土豆、红薯、杂豆、玉米、燕麦、小米等）	①	②	③	④	⑤
（2）**水果**	①	②	③	④	⑤
（3）**蔬菜、菌藻类**（蘑菇、海带等）	①	②	③	④	⑤
（4）**奶制品**（牛奶、酸奶、奶粉或奶酪，不包括酸酸乳等）	①	②	③	④	⑤
（5）**豆制品**（豆腐、豆浆、豆干、豆腐丝、腐竹、豆花等）	①	②	③	④	⑤
（6）**含糖饮料**（含糖碳酸饮料、含糖茶饮料、市售果蔬汁饮料、乳饮料、运动饮料等）	①	②	③	④	⑤
（7）**早餐**	①	②	③	④	⑤
（8）**油炸食品**（油炸方便面、油条、炸串、薯片等）	①	②	③	④	⑤
（9）**西式快餐**（汉堡、薯条、炸鸡等）	①	②	③	④	⑤

4. 下列农作物和食物对应**错误**的是？
①土豆和薯条　②小麦和馒头　③红薯和粉条　④高粱和爆米花　⑤水稻和大米

5. **连一连**：参考示例，将左边的食物和右边的食物分类正确连线

土豆	蔬菜水果类
鲤鱼	谷薯杂豆类
冰糖	大豆类和坚果
西兰花	纯能量食物
黄豆豆浆	动物性食物

6. （**三~六年级跳过此题**）以下食物及其富含的营养素对应**错误**的是：
①动物肝脏—维生素 A　②米饭—碳水化合物　③桔子—维生素 C　④鸡肉—钙

7. 数一数你**昨天**吃了多少种食物？（例如芹菜牛肉包子里面可能含面粉、牛肉、芹菜 3 种食物，调味料除外）
① 0~3 种　② 4~7 种　③ 8~11 种　④ 12 种及以上

8. 你认为以下哪种是比较健康的零食？
①薯片　②蜜枣　③酸奶　④奶茶　⑤蛋糕

9. **上周**你有几天的**累计锻炼时间超过 60 分钟**？（把**一天内的运动时间加起来**，包括体育课运动、课间操、跑步、快走、游泳等使心跳明显加快、有点吃力的活动）

上学日（周一至周五）：① 0 天　② 1 天　③ 2 天　④ 3 天　⑤ 4 天　⑥ 5 天

周末（周六日）：① 0 天　② 1 天　③ 2 天

10. **上周**你平均每天有多长时间是**在看电子屏幕**？（课外时间使用电视、手机、电脑、pad、游戏机等）

上学日（周一至周五）：①<1 小时　②1~2 小时　③3~4 小时　④5~6 小时　⑤≥7 小时

周末（周六日）：①<1 小时　②1~2 小时　③3~4 小时　④5~6 小时　⑤≥7 小时

11. （1）你最近一次测**身高**为_______厘米，**体重**为______公斤（或_______斤）；____年____月测量

（2）你觉得自己的**体型**怎样？　①偏瘦　②适中　③偏胖

12. （**三四年级跳过此题**）BMI（体质指数）是用来评价体型的常用方法，公式：**BMI = 体重(kg) / 身高 ^{2}(m^2)**
小兰 8 岁整，体重为 28.8kg，身高为 1.20m，已知 8 岁女孩 BMI 超过 19.4 kg/m^2 属于肥胖，你来判断一下她是否肥胖呢？
①是　②不是　③不清楚

13. （1）假如今天是 2019 年 6 月 1 日，你想喝牛奶，在家里找到了这三种，你会选择哪一个？

①	②	③
纯牛奶	鲜牛奶	高钙奶
生产日期：2019年5月20日	生产日期：2019年5月26日	生产日期：2019年4月29日
保 质 期：室温14天	保 质 期：室温3天	保 质 期：室温30天

（2）有以下两个品种的牛奶，观察它们的营养成分表，你觉得**哪个品种更适合超重肥胖的人**？

① 品种 1　　②品种 2　　③不清楚

品种 1 营养成分表

项目	每100毫升	营养素参考值%/NRV%
能量	265千焦（kJ）	3%
蛋白质	3.5克（g）	6%
脂肪	3.4克（g）	6%
碳水化合物	4.7克（g）	2%
钠	40毫克（mg）	2%
钙	122毫克（mg）	15%

品种 2 营养成分表

项目	每100毫升	营养素参考值%/NRV%
能量	182千焦（kJ）	2%
蛋白质	3.0克（g）	5%
脂肪	1.3克（g）	2%
碳水化合物	4.9克（g）	2%
钠	72毫克（mg）	4%
钙	125毫克（mg）	16%

（3）（**三四年级跳过此题**）上题的食品营养标签中，**"营养素参考值%/NRV%"**是指 100 克/毫升该食品所含营养成分占每日参考摄入量的**百分比**。那么，如果只喝靠**品种 2** 的牛奶来摄入钙，那喝多少就可以满足我们一天需要的**钙**？（估算即可）

① 200 毫升左右　② 400 毫升左右　③600 毫升左右　④不清楚怎么算

14. 中国居民**平衡膳食宝塔最底下的一层**是什么？
①蔬菜水果　②动物性食物　③谷薯类　④奶豆类　⑤不清楚

15. **（三四年级跳过此题）**中国居民膳食指南建议每天吃 **200~350 克的水果**，以下哪个选项**不能**达到推荐量？（图中 330 毫升易拉罐为参照物）

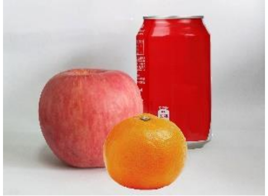
①一个苹果和一只桔子

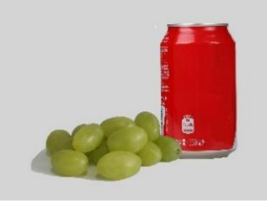
②15 颗葡萄

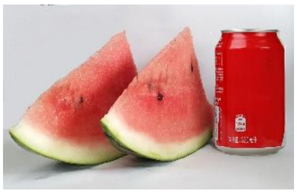
③两块西瓜

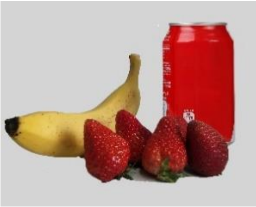
④一根香蕉和五颗草莓

16. 如果同学想约你去吃**路边摊烧烤**，周边环境并不卫生，你会怎么做？
①既然同学邀请，就吃一次没问题，还是去吧　② 烧烤那么好吃，当然要吃了
③劝说朋友去吃别的，路边烧烤不健康不卫生　④借口有事不去

17. 假如你的家人患有较严重的糖尿病，听别人劝说正准备停药吃保健品，你会怎么做？
①仔细阅读保健品说明书或咨询商家，看是否适合家人　②吃药有副作用，而保健品没有，家人应该听劝
③建议家人咨询医生、营养师或其他专业人士再决定　④我不懂，不给建议
