# Supplementary material for: Development and validation of a food and nutrition literacy questionnaire for Chinese school-age children
Source: PLoS One. 2021 Jan 6;16(1):e0244197. doi: 10.1371/journal.pone.0244197 (PMC7787443; doi:10.1371/journal.pone.0244197)
Supplement: S2 File — (PDF) [file pone.0244197.s004.pdf]

Food and Nutrition Literacy Questionnaire for Chinese School-age Children (FNLQ-SC)

1. Choose the corresponding answer according to your situation (√).

Items	Never	Seldom	Sometimes	Usually	Always
(1)I am concerned about nutritive value when I select food.	①	②	③	④	⑤
(2)I decide what food to eat by myself.	①	②	③	④	⑤
(3)I am interested in Chinese food culture.	①	②	③	④	⑤
(4)I can make some Chinese traditional food (such as dumpling, zongzi, and spring roll.)	①	②	③	④	⑤
(5)I abide by table manners (the elder first, no noise, no messing with meals, help tidying up tableware, etc.)	①	②	③	④	⑤
(6)I fill/buy as much food as I can eat up and seldom waste it.	①	②	③	④	⑤
(7)I help cooking.	①	②	③	④	⑤
(8)I wash my hands before eating.	①	②	③	④	⑤
(9)I am picky for more than two kinds of food.	①	②	③	④	⑤
(10)I am influenced by food advertisements on TV or the Internet when I choose food.	①	②	③	④	⑤
(11)I am concerned about nutrition and health information.	①	②	③	④	⑤
(12)I actively discuss the nutrition and health information with others.	①	②	③	④	⑤
(13)I am concerned about the food label on the package when I buy food.	①	②	③	④	⑤

2. Do you agree with the following statements?

Items	Strongly disagree	Disagree	Neither agree nor disagree	Agree	Strongly agree
(1) The eating pace has little effect on the health.	①	②	③	④	⑤
(2) You don't need to eat breakfast if you're not hungry.	①	②	③	④	⑤
(3) You don't need to drink water when you're not thirsty.	①	②	③	④	⑤
(4) It double benefit to watch a video while eating.	①	②	③	④	⑤
(5) Fruit wine is not alcoholic beverage.	①	②	③	④	⑤
(6) Children need to eat much meat to keep growing.	①	②	③	④	⑤
(7) The expired bread for one day could be eaten, if it looks well.	①	②	③	④	⑤
(8) Leftover food should be thoroughly heated before eating.	①	②	③	④	⑤
(9) Leftover food should be stored in the refrigerator in time, without cooling.	①	②	③	④	⑤
(10) The chopping board could be used to cut cooked meat after washing, which has just been used to cut raw meat.	①	②	③	④	⑤

3. **<u>During the past 7 days</u>**, how many days did you eat the following foods? (Choose the corresponding answer according to your situation using symbol √).

**Foods**	**0 day**	**1~2 days**	**3~4 days**	**5~6 days**	**7 days**
(1)**Coarse grains** (potatoes, lentils and legumes, corn, oats, etc.)	①	②	③	④	⑤
(2)**Fruits**	①	②	③	④	⑤
(3)**Vegetables, including fungi and algae**	①	②	③	④	⑤
(4)**Dairy products**	①	②	③	④	⑤
(5)**Soy products** (tofu, soy milk, bean curd, etc.)	①	②	③	④	⑤
(6)**Sugar-sweetened beverages**	①	②	③	④	⑤
(7)**Breakfast**	①	②	③	④	⑤
(8)**Fried food**	①	②	③	④	⑤
(9)**Western fast food**	①	②	③	④	⑤

4. Which match of crop and processed food is wrong?
①Potato and chip ②Wheat and bun ③Sweet potato and vermicelli
④Sorghum and popcorn ⑤Paddy and rice

5. Link correctly referring to the example.

Potatoes	Vegetables and fruits
Carp	Grains and tubers
Rock candy	Soybeans and nuts
Broccoli	Energy-source foods
Soybean Milk	Animal foods

6. Which of the match (foods and their rich in nutrients) is wrong? <u>(Skip this question for the 3rd to 6th grader)</u>
①Animal liver-vitamin A ②Rice-carbohydrate
③Orange-vitamin C ④Chicken-calcium

7. Yesterday, how many kinds of foods did you eat?
①0~3 ②4~7 ③8~11 ④≥12

8. Which of the following snacks is healthier?
①Potato chips ②Candied date ③Yogurt ④Milk tea ⑤Cake

9. During the past 7 days, on how many days were you physically active for a total of at least 60 minutes per day? (Add up all the time you spent in any kind of physical activity that increased your heart rate and made you breathe hard some of the time.)
<u>School day (Monday to Friday):</u> ①0 day ②1 day ③2 days
④3 days ⑤4 days ⑥5 days
<u>Weekend (Saturday and Sunday):</u> ①0 day ②1 day ③2 days

10. During the past 7 days, how many hours do you play video or computer games or use a computer for something that is not school work?
<u>School day (Monday to Friday):</u> ①<1hour ②1~2hours ③3~4hours
④5~6hours ⑤≥7hours
<u>Weekend (Saturday and Sunday):</u> ①<1hour ②1~2hours ③3~4hours
④5~6hours ⑤≥7hours

11. (1) Fill in your height: _______ cm, and weight: ______ kg;
The date of examination: ________.
(2) How do you think about your weight status?

①Wasted ②Normal ③Overweight

12. Body Mass Index (BMI) index is often used to assess weight status, which is defined as weight(kg) / height(m^2). (Skipping this question for the 3rd to 4th grader.)
Lisa is 8 years, with the height of 1.20 m and weight of 28.8 kg. It is known that for an 8-year-old girl, the obesity is diagnosed with the BMI more than 19.4 kg/m^2. Please calculate Lisa's BMI and judge if she is obese?
①Lisa is obese. ②Lisa is not obese. ③I am not sure.

13. (1) Today is June 1, 2019, and which milk would you choose?

①	②	③
Pure milk	Fresh milk	High calcium milk
**Production date:** 2019.5.20	**Production date:** 2019.5.26	**Production date:** 2019.4.29
**Shelf life:** 14 days	**Shelf life:** 3 days	**Shelf life:** 30 days

(2) Which milk is suitable for overweight person? Referring to the nutritional label.

①Milk 1

Nutrients	Per 100 ml	NRV%
**Energy**	265 kJ	3%
**Protein**	3.5 g	6%
**Fat**	3.4 g	6%
**Carbohydrate**	4.7 g	2%
**Sodium**	40 mg	2%
**Calcium**	122 mg	15%

②Milk 2

Nutrients	Per 100 ml	NRV%
**Energy**	182 kJ	2%
**Protein**	3.0 g	5%
**Fat**	1.3 g	2%
**Carbohydrate**	4.9 g	2%
**Sodium**	72 mg	4%
**Calcium**	125 mg	16%

(3) In the above nutritional label, "NRV%" means the percentage of the nutrients in 100 ml milk accounting for daily reference intake. How much milk 2 one person need to intake to meet his/her calcium requirement? (Skipping this question for the 3rd and 4th grader)
① 200 ml ② 400 ml ③ 600 ml ④ Not sure

14. What foods is it in the bottom layer of "Chinese food guide pyramid"?
①Vegetables and fruits ②Animal food ③Grains and tubers
④Dairy and beans ⑤Not sure

15. The Chinese dietary guideline recommends 200-350 g of fruits every day. Which of the following options cannot reach the recommended amount? (The 330 ml can in the pictures is a reference object)

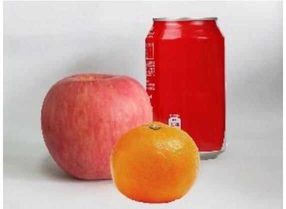

①An apple and an orange

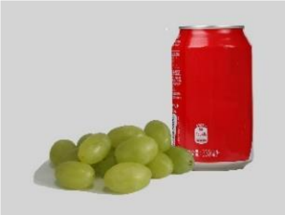

②15 grapes

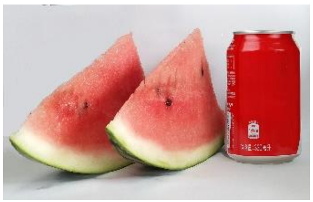

③Two pieces of watermelon

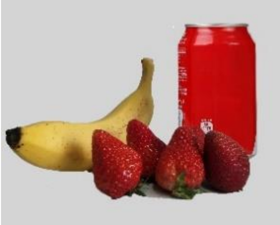

④One banana and five strawberries

16. Your friend invites you to eat barbecue, but the restaurant's environment is not hygienic. What would you do?
①Just one dinner doesn't matter.
②I don't care the environment and the barbecue is so delicious.
③I will persuade my friend to another restaurant or other foods, because the unsafe foods might cause food-borne diseases.
④I will make an excuse to refuse the invitation.

17. If your family member suffers from diabetes, and he/she is going to take some "healthy food" of advertisement, instead of medicine. What would you do?
①I will read the instructions of the product carefully or consult the salesman to judge if it is suitable for my family member.
②It is correct to take the healthy food instead of medicine, because the medicine has more side effects than healthy foods.
③I will suggest him/her to consult a doctor, dietitian or other professional persons before making the decision.
④I don't know anything about it, so I won't give any advice.
